# Introduction to the New Editorial Series, *Notes of Allergy Watchers*

**DOI:** 10.1097/WOX.0b013e3181a5d3b4

**Published:** 2009-05-15

**Authors:** Lanny J Rosenwasser

**Affiliations:** 1 Editor-in-Chief, World Allergy Organization Journal

## 

It is my great pleasure to introduce our series, *Notes of Allergy Watchers*, with a very timely book review on the autobiography of Professor Alain de Weck. The new series will cover viewpoints and recollections concerning the science and practice of allergy and immunology. Authors will provide a look backward and also observations and speculations concerning the science of our discipline and projections for potential changes within our field in the future.

It is also timely and appropriate that Professor Johannes Ring, our Executive Editor and the prior Editor in Chief, provides this review in the first of the series. Professor Alain de Weck is a titan in our field; he has been involved in allergy and immunology for over 60 years and has experienced the growth of our discipline both clinically and scientifically from its humble beginnings to its present state. Trained by Doctor Herman Eisen at Washington University in St. Louis in basic immunology, and then extending his rigorous scientific background to clinical and translational issues and to basic science, Alain de Weck's experience has been broad and illuminating. He has been an integral part of a variety of organizations in academic societies that have had a tremendous effect on our current organization including the World Allergy Organization.

Alain de Weck has been an important contributor in a wide variety of venues, and his perspective has been quite broad and entertaining. This review by Johannes Ring expertly identifies the breadth of Alain de Weck's contributions, and it is a pleasure to have this review and our distinguished colleagues, Professors Ring and de Weck, highlighted in the inaugural *Notes of Allergy Watchers*.

Lanny J. Rosenwasser, MD

(Editor-in-Chief)

World Allergy Organization Journal

