# Introduction to the New Editorial Series, *Notes of Allergy Watchers*: Erratum

**DOI:** 10.1097/WOX.0b013e3181b0e428

**Published:** 2009-06-15

**Authors:** Herman Eisen

## 

In the article "Introduction to the New Editorial Series, *Notes of Allergy Watchers*", which appeared in volume 2 of *The World Allergy Organization Journal *on page 61, the name Doctor Arthur Eisen is incorrect. This should appear as Doctor Herman Eisen.

This error has been noted in the original version of the article, which is available at http://www.waojournal.org.
